# Clinical Practice Guidelines on the Management of Menopause in Malaysia 2022: A review from a primary care perspective

**DOI:** 10.51866/cpg.910

**Published:** 2025-07-22

**Authors:** Wai Khew Lee, Noreen Zhi Min Ooi, Soo Lee Goh

**Affiliations:** 1 MBBS, MMed (Fam Med), MSc., Department of Primary Care Medicine, University Malaya, Kuala Lumpur, Malaysia. Email: leewaikhew@gmail.com; 2 MBChB, MMed (Fam Med), Klinik Kesihatan Sentul, Kuala Lumpur, Wilayah Persekutuan, Malaysia.; 3 MD, MMed (Fam Med), Klinik Kesihatan Masjid Tanah, Masjid Tanah, Melaka, Melaka, Malaysia.

**Keywords:** Menopause, Practice guidelines, Appraisal, AGREE tools, Primary care

## Abstract

**Introduction:**

Clinical practice guidelines (CPGs) are important tools that assist primary care doctors in their daily practice. Menopause is a physiological condition that affects all women, and the latest Malaysian CPG on menopause can provide guidance on the optimal management of women facing this condition. However, not all CPGs are of acceptable quality, and it is important that primary care doctors learn to critically appraise CPGs to ensure their practice is evidence-based and relevant to their patients. This review aimed to critically appraise the Clinical Practice Guidelines on the Management of Menopause in Malaysia 2022 to ensure that it is valid and applicable to primary care in Malaysia.

**Methods:**

Three family medicine specialists independently appraised the CPG using the AGREE II Instrument, which consists of six domains assessing the following: (1) scope and purpose, (2) stakeholder involvement, (3) rigour of development, (4) clarity and presentation, (5) applicability and (6) editorial independence. They also provided an overall assessment of quality and recommendation on the use of the CPG following the AGREE II guideline.

**Results:**

The CPG scored >70%, which is the threshold of quality in all domains except for applicability. The reviewers deemed the CPG to be of high quality and recommended its adoption without any modifications.

**Discussion:**

It is important to ensure that recommendations in a CPG are evidence-based and valid before they are adopted into practice. The AGREE II Instrument can assist primary care doctors in critically appraising CPGs.

## Introduction

Menopause is a normal biological process that all women will undergo. It is marked by permanent cessation of menstruation when ovarian follicular activity ceases. A woman is said to have reached menopause when there is no menstruation for at least 12 months.^1^ The average age for Malaysian women to attain menopause is 50.7 years.^2^ However, in some women, menopause comes too early and is defined as premature menopause when it happens before the age of 40 years. Premature menopause has been shown to be associated with significant cardiovascular events.^3^ Although menopause is considered a natural phenomenon, there are associated symptoms called perimenopausal symptoms, which range from mild to distressing, as shown in some local studies.^4,5^ In addition, the risks of other conditions, such as osteoporosis and cardiovascular disease, increase after menopause.^5^ The average life expectancy of a Malaysian woman is 77.8 years, which means most women will live in a state of menopause for almost 30 years in the latter part of their lives.^6^ It behoves primary care doctors not only to understand this condition well but also to provide appropriate management to help ensure a good quality of life for these women in their golden years. However, the provision of care specifically addressing menopause is far from satisfactory in Malaysia. A systematic review in a primary care setting found that only 50% of postmenopausal women were screened for and diagnosed with osteopenia or osteoporosis.^7^ Another recent study conducted in three states in Malaysia showed a less than 1% prescription rate for menopausal hormone therapy (MHT) among primary care doctors in government clinics.^8^

In the busyness of daily practice, clinical practice guidelines (CPGs) and their summarised key recommendations remain the go-to tool for primary care doctors to provide evidence-based management for their patients. The availability of local CPGs provides the assurance that recommendations are relevant to their practice. However, they still need to be assured that guidelines adhere to the accepted standards of practice. Most primary care doctors lack training in critically appraising CPGs.

This review aimed to assist primary care doctors in determining the validity and applicability of the recommendations in the Clinical Practice Guidelines for the Management of Menopause in Malaysia 2022 for their patients in their busy day-to-day practice.

## Methods

The reviewers consisted of three family medicine specialists with 3-26 years of experience working in government health clinics. We used the AGREE II Instrument, which is an internationally accepted critical appraisal tool, to review the CPG.^9^ This critical appraisal tool has 23 items grouped into six domains: (1) scope and purpose, (2) stakeholder involvement, (3) rigour of development, (4) clarity and presentation, (5) applicability and (6) editorial independence. Each item is scored on a Likert scale ranging from 1 to 7, with *strongly disagree* scored as 1 and *strongly agree* scored as 7. The three reviewers independently appraised the CPGs using this checklist. The standardised domain scores were calculated according to AGREE II. Any domain that had a standardised score of >70% fulfilled the good quality threshold. The reviewers then met virtually to discuss the findings. Any disagreements were clarified, and only domains that scored <70% were re-scored. The standardised domain scores before and after discussion were tabulated for comparison.

There were two final questions on the overall assessment of the quality and whether the reviewers will recommend the use of the CPG with or without modifications or reject the CPG based on their judgement.

## Findings

All reviewers found the CPG well-written with a good layout for ease of reference. The sections were well-labelled. The recommendations were framed using the GRADE format, and the level of evidence for the recommendations followed the Canadian Task Force on Preventive Care outline.^10,11^ Table 1 shows the standardised domain scores calculated independently before and after discussion among the reviewers. Only Domain 5: applicability scored <70% in the initial independent appraisal. Although the scores increased after discussion, applicability remained the only domain that did not achieve the quality threshold set by the reviewers.

**Table 1:** Standardised scores for all domains before and after discussion.

Domain		Pre	Post
1	Scope and purpose	100%	100%
2	Stakeholder involvement	79.60%	79.60%
3	Rigour of development	76.40%	79.20%
4	Clarity and presentation	100.00%	100%
5	Applicability	56.90%	61.11%
6	Editorial independence	100.00%	100.00%

### Domain 1: Scope and purpose

This domain received unanimous full marks from all three reviewers. The objective of the CPG to provide guidance for the management of menopause was clearly stated, and the treatment population was clearly identified to include perimenopausal, menopausal and postmenopausal women. The target healthcare users were also identified, and the settings included primary, secondary and tertiary settings.

### Domain 2: Stakeholder involvement

Although this domain scored >70%, the reviewers were concerned regarding the lack of participation of the patient population in the development of the CPG. The main developers of the CPG were obstetrics & gynaecology (O&G) specialists and one endocrinologist. No primary care physician was included in the development group, although one primary care doctor, along with other healthcare professionals including a pharmacist, an occupational health doctor, a midwife and other O&G specialists working in various settings, was in the review panel. It has been shown that involvement of patients or their representatives in CPG development leads to better adoption of CPGs because it takes into account patients’ preferences and experiences with the disease, social circumstances and habits as well as the cultural context.^12-14^ The reviewers noted that the CPG explored some of these aspects through a literature review that looked into local women’s perspective and experience of menopause.

### Domain 3: Rigour of development

This domain assesses the details of the development process, which could affect the overall quality of CPGs. There are eight items in this domain. The search strategy was clear and systematic, with the criteria for selection of evidence well-described. However, the reviewers could not find any documentation of the strength and limitation of the body of evidence in the CPGs, which contributed largely to the lower score obtained. In addition, the side effects and risks were mainly described for the MHT only. Nonetheless, the methods for formulating the recommendations and the link between the supporting evidence and the recommendations were outlined clearly. Two well-known international specialists were invited as the external reviewers, which enhanced the quality of the CPG. The authors also outlined the procedure on how to update the CPG clearly.

### Domain 4: Clarity of presentation

This domain scored maximum points among all reviewers. The key recommendations were arranged in colour-coded boxes, which allowed ease of identification. The recommendations were specific, and options were clearly outlined for specific populations and situations. For example, the use of MHT therapy was presented in a separate section. Its indications, contraindications and special precautions, follow-up and side effects with different preparations were highlighted for different populations of women (e.g. women with an intact uterus, women who have undergone hysterectomies and cancer survivors) and supported with evidence. The concerns of many women regarding the effects of MHT on cardiovascular disease and cancer were also addressed.

### Domain 5: Applicability

Applicability was the only domain that scored below the threshold of quality set prior to the discussion. Two reviewers felt that there was not enough discussion or clarity on the management of menopause in the primary care setting, although it was mentioned that any doctors who have sufficient training should be able to manage this condition and that it could be used in the primary care setting. Most women have access to their primary care doctors and are comfortable to discuss their health issues with them. Many would find it cumbersome to be referred to the hospital just to obtain their treatment. However, there are many challenges and barriers especially in government health clinics, where many of the population obtain their healthcare. Access to appropriate MHT is still a substantial constraint, as most formulations can only be prescribed by O&G specialists in the government formulary. Many primary care doctors are also not well-trained and find it uncomfortable to discuss this normal physiological phenomenon with their patients.

The CPG scored low in terms of lack of discussion on facilitators and barriers as well as advice on how to put the recommendations into practice. There was no discussion on the potential resource implications of the recommendations. The monitoring recommended for auditing was also limited to looking into the side effects of MHT.

### Domain 6: Editorial independence

The evidence of editorial independence was clearly outlined. Although the development of the CPG received some external funding, this was fully disclosed by the authors. In addition, all authors filled out a disclosure form and declared that none held any interests in the funding organisations. There was an explicit statement specifying that the views and interests of the funding organisation did not affect the final recommendations of the CPG.

### Overall guideline assessment

All three reviewers rated the CPG 6/7 in terms of quality and would recommend it to be used by primary care doctors without modifications. They commended that the CPG has a section on views from the major different religious authorities prevalent in Malaysia. In the current society, where most people still profess that religion is important to them, the inclusion of this section will guide primary care doctors in their discussion when counselling patients on the best options for managing menopause.

### Key recommendations of the CPG

There is a long list of key recommendations in the CPG. In summary, any woman approaching menopause should be managed holistically; preventive screening for other associated conditions (e.g. osteoporosis and breast cancer) should be carried out simultaneously. MHT is effective and safe overall, backed by evidence, and is recommended for most women. However, treatment should be individualised, and a thorough discussion and examination should be conducted first. It is recommended that healthcare practitioners receive training before managing these women. Alternative therapies are also available for women who are unable or unwilling to receive MHT.

### How can the CPG be applied in primary care?

While there were not many details on how to use the CPG in primary care, we believe that the recommendations are sound and can be applied to menopausal women seen by their primary care doctors. The way forward is to use the CPG as the foundation to create a training module adjusted to the primary care setting. This can be taken up by the Ministry of Health (MOH) or any professional primary care organisation. In addition, the wide range of MHT should be made available for prescription by family medicine specialists and medical officers in the MOH to ensure that the key recommendations of the CPG can be implemented, as most women in the community seek healthcare in government health clinics. It is hoped that the O&G fraternity would also tap into the expertise of family medicine specialists to work together to ensure that the CPGs can be adopted easily and widely.

Although this review was conducted by family medicine specialists working in the MOH, we believe that the findings are equally applicable to primary care doctors working in the private sector. The challenges may be different, with the costs of MHT being a significant concern, as most expenses are borne by patients themselves.

## Case discussion

Below, we showcase two typical cases seen in primary care and the application of the CPG for each of them.

### Case scenario 1

Madam S is a 48-year-old woman with three children. She has no significant medical history, apart from a lower-segment caesarean section 12 years ago for foetal distress. Both of her parents have type 2 diabetes mellitus and hypertension. There is no personal or family history of cancer.

She initially sought medical attention for symptoms of dysuria, lower abdominal discomfort and a sensation of incomplete bladder emptying. She was treated with urine alkaliser and a course of antibiotics but showed no improvement. Investigations for urinary tract infection (i.e. urinalysis and midstream urine for culture and sensitivity) yielded negative findings.

At her last follow-up visit, she revealed that she was experiencing perimenopausal symptoms, including hot flushes, decreased libido and discomfort during intercourse. Her menstrual cycles had become shorter and lighter. The most disturbing symptoms for her were genitourinary symptoms, whereas she was less bothered by vasomotor symptoms. Madam S managed her vasomotor symptoms by using conventional methods such as switching on their air-conditioning and drinking cold drinks.

Cardiovascular risk assessment using the Framingham score showed a low 10-year risk. Based on her personal and family history, she had a low risk of cancer. Based on her Osteoporosis Self-Assessment Tool for Asians results, she had a low risk of osteoporosis. She was offered mammogram for breast cancer screening, as she had not undergone any before, and her cervical cancer screening was up to date, where she recently had a human papilloma virus test.

According to the CPG, topical vaginal oestrogen cream is recommended for the management of genitourinary symptoms. For topical application, additional progestogen is not needed for endometrial protection. However, topical oestrogen preparations do not offer additional benefit for the treatment of hot flushes or prevention of osteoporosis, heart disease or other major health conditions.

If Madam S was unable to cope with her vasomotor symptoms, she can be offered oral MHT containing oestrogen and progestogen, as she has an intact uterus. Prior to initiating oral MHT, her risk for venous thromboembolism, cardiovascular events, breast cancer and liver disease should be assessed.

She was offered MHT to relieve her symptoms. A low-dose vaginal oestrogen cream was prescribed, which improved her condition after 2 weeks of use.

### Case scenario 2

Madam ZB is a 50-year-old woman with five children. She has underlying controlled hypertension, dyslipidaemia and type 2 diabetes mellitus. She has no personal or family history of cancer or breast lumps. Her elderly mother had a history of right hip fracture, which required total hip replacement at the age of 70 years.

Madam ZB came for a routine follow-up of her non-communicable disease. However, she complained of having difficulty sleeping and hot flushes for the past 3 months. She felt significantly hot and disturbed even when sleeping in an air-conditioned room at night. She also found herself becoming easily upset and tearful for no apparent reason with mood swings. Her menstrual cycles were irregular for the last 6 months. Her last menstrual period was 2 months ago. As her husband is working outstation, and she had not been sexually active for the past 3 months, the possibility of pregnancy was low. She did not have any weight loss or drop in appetite.

Her physical examination findings were unremarkable. Blood investigations showed normal blood test results, with normal thyroid function. She underwent Pap smear for cervical cancer screening last year, which revealed normal findings.

She was advised lifestyle modification and offered MHT to relieve her symptoms. She was also arranged for a mammogram appointment to screen for breast cancer and a bone densitometry (BMD) scan to screen for osteoporosis. She was apprehensive about starting MHT and wanted to try some alternative therapy first. She was happy with the cessation of her menstruation and was not keen to have the hassle of monthly bleeding, which would affect her religious obligations. After 1 months, her symptoms were not relieved but had worsened. Her BMD showed osteopenia in the spine and hip. She was counselled again on MHT, and her concerns regarding MHT were elicited and addressed.

In this case, the indication for MHT was to relieve the vasomotor symptoms and mood disturbances as well as prevent osteoporosis. As she was still in the perimenopausal state and has an intact uterus, the CPG recommend the use of combined oestrogen with progestogen as the MHT of choice. The small increased risk of breast cancer, which is related to the duration of MHT and type of progestogen used, was explained to the patient. She understood the risks and was agreeable to the MHT. Daily combined oestradiol valerate 2 mg and norgestrel 500 mcg, which were a combined cyclical MHT, were started for her, as this preparation would cause less irregular bleeding, with the plan to switch to combined continuous preparations after 1 year. In the follow-up during the subsequent month, her symptoms improved tremendously, which made Madam ZB happy.

The two case discussions above illustrate the application of the CPG in the daily practice of primary care doctors. The selection of MHT needs to be tailored to the woman, taking into consideration symptoms that are affecting her quality of life, personal health risks and preferences, age and/or time since menopause in relation to the timing of starting MHT.

## Conclusion

The Clinical Practice Guidelines on the Management of Menopause in Malaysia 2022 fulfils almost all the criteria of AGREE II and is of high quality. The key recommendations should be adopted by primary care doctors and applied to their patients who are experiencing this phase of their lives. Barriers to implementation should be identified and tackled at both institutional and personal levels to ensure all women receive the best care possible during their golden years.
